# Effect of Atmospheric-Pressure Plasma Treatments on Fracture Toughness of Carbon Fibers-Reinforced Composites

**DOI:** 10.3390/molecules26123698

**Published:** 2021-06-17

**Authors:** Won-Jong Kim, Young-Jung Heo, Jong-Hoon Lee, Kyong Yop Rhee, Soo-Jin Park

**Affiliations:** 1 Department of Chemistry, Inha University, 100 Inharo, Incheon 22212, Korea; wonjongkim@inha.edu (W.-J.K.); heoyj1211@naver.com (Y.-J.H.); boy834@naver.com (J.-H.L.); 2 Department of Mechanical Engineering, College of Engineering, Kyung Hee University, Yongin 17104, Korea

**Keywords:** polymer–matrix composites (PMCs), fracture toughness, surface properties

## Abstract

In this study, nano-scale fillers are added to epoxy matrix-based carbon fibers-reinforced composites (CFRPs) to improve the mechanical properties of multi-scale composites. Single-walled carbon nanotubes (SWCNTs) used as nano-scale fillers are treated with atmospheric-pressure plasma to introduce oxygen functional groups on the fillers’ surface to increase the surface free energy and polar component, which relates to the mechanical properties of multi-scale composites. In addition, the effect of dispersibility was analyzed through the fracture surfaces of multi-scale composites containing atmospheric-pressure plasma-treated SWCNTs (P-SWCNTs) under high load conditions. The fillers content has an optimum weight percent load at 0.5 wt.% and the fracture toughness (*K_IC_*) method is used to demonstrate an improvement in mechanical properties. Here, *K_IC_* was calculated by three equations based on different models and we analyzed the correlation between mechanical properties and surface treatment. Compared to the composites of untreated SWCNTs, the *K_IC_* value is improved by 23.7%, suggesting improved mechanical properties by introducing selective functional groups through surface control technology to improve interfacial interactions within multi-scale composites.

## 1. Introduction

In recent decades, carbon fibers-reinforced composites (CFRPs) have been widely used in many industries such as aerospace design, automotive, and sports, due to their high fatigue strength, corrosion resistance, and good coefficient of thermal expansion [1,2,3,4,5]. One of the factors that enhance the excellent properties mentioned above is the interfacial interaction between the carbon fibers (CFs) and the epoxy matrix in CFRPs [6,7]. It has a chemically stable molecular structure due to its high strength and cross-linked density, which are inherent properties of epoxy resins. However, this means that the resistance to impact resistance, crack propagation, and brittleness is insufficient [8,9,10,11]. In addition, problems in the area of critical cracking are limited in various industrial applications [12,13,14,15]. To overcome the mechanical property problems mentioned above, there are many studies that improve the interfacial adhesion by adding nano-scale fillers between the CFs and the epoxy matrix [16,17,18]. Recently, many experts have confirmed that the addition of carbon nanotubes (CNTs) [19], carbon black [20], graphene [21], and nano-silica [22] to the epoxy matrix improves the mechanical properties. Nano-scale CNTs have proven to be an excellent material for activating both the CFs and the surface of the matrix [23,24,25]. CNTs are classified into two types according to their structural properties: single-walled carbon nanotubes (SWCNTs) and multi-walled carbon nanotubes (MWCNTs). In particular, SWCNTs are expected to act as high structural and functional elements in next-generation composites because of their superior elastic deformation, fracture retention, and lightweight properties compared to conventional composites fillers [26,27,28]. However, SWCNTs have a short diameter of about 15 to 30 nm and are difficult to distribute uniformly and efficiently because of the inherent properties of the van der Waals forces acting on the outer diameter of the fillers, which promotes aggregation [29,30]. This means that in multi-scale composites, the aggregation of SWCNTs can interfere with interfacial interactions and degrade mechanical properties.

Recent studies are trying to overcome this problem by chemically modifying the surface of CNTs. Modifying the surface of CNTs to alter mechanical and chemical properties can be achieved through many treatment methods, including atmospheric-pressure plasma [31], electron beams [32], γ-rays [33], ozone [34], and corona [35]. In this work, a promising method is used to selectively attach various functional groups, while deforming the surface in the desired direction depending on temperature, current, voltage, gas atmosphere, while still maintaining the inherent properties of the CNTs [36]. Functional groups activated through atmospheric-pressure plasma treatment increase the formation of hydrophilic groups on the surface of the CNTs and are directly related to the increase in surface free energy. On the other hand, oxygen treatments have the advantage of increasing the interfacial adhesion and wetting properties of the fiber while attaching various functional groups such as carboxyl (–COOH), carbonyl (–CO), and hydroxyl groups (–OH) [37,38]. This can be interpreted as a method to enable better interfacial adhesion and interactions between the CNTs and the epoxy matrix of multi-scale composites, providing excellent mechanical properties [39,40,41].

In this work, the effect of atmospheric-pressure plasma-treated SWCNTs (P-SWCNTs) on the interfacial properties of multi-scale composites was investigated. To accurately analyze the mechanical properties according to the interfacial properties, neat composites and untreated composites were compared by weight content. In addition, the chemical composition and morphological structure changes of P-SWCNTs are discussed in detail.

## 2. Results and Discussion

### 2.1. Characterization of Nano-Scale Fillers

**Figure 1:**
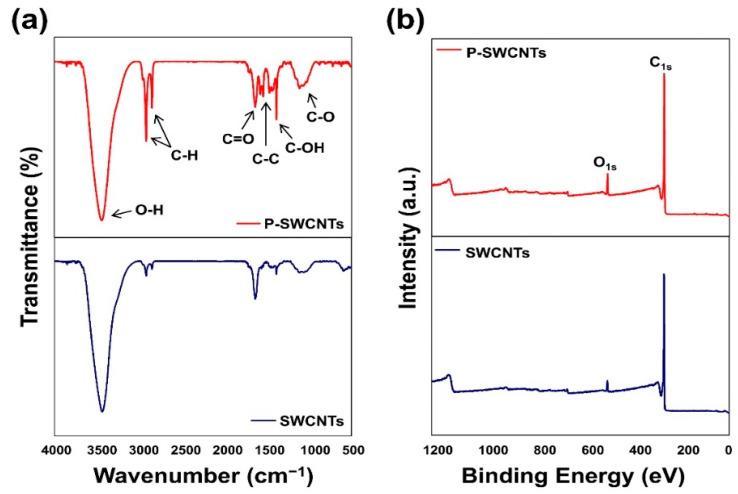
(**a**) FT-IR and (**b**) XPS spectra of SWCNTs and P-SWCNTs.

Figure 1a shows the FT-IR spectra of SWCNTs and P-SWCNTs and it can be seen that peaks occurred near 1114, 1382, 1751, 2920, and 3436 cm^−1^. For the SWCNTs, differences after atmospheric-pressure plasma treatment were observed as larger peaks at wavenumbers 1699 and 1385 cm^−1^, which correspond to the carboxyl (–COOH) stretching vibrations of C=O and C–OH, respectively, while the peaks at 1110 and 2918 cm^−1^ correspond to the stretching vibrations of C–O and C–H, respectively [42,43].

The XPS spectra on the surface of SWCNTs and P-SWCNTs, as well as the atomic content concentrations of C and O, are shown in Figure 1b and Table S1 (Supplementary Materials), respectively. It was found that the C_1s_/O_1s_ ratios of the SWCNTs and P-SWCNTs samples were 0.04% and 0.09%, respectively. This is because the atmospheric-pressure plasma treatment of the SWCNTs surface generates oxygen functional groups.

**Figure 2:**
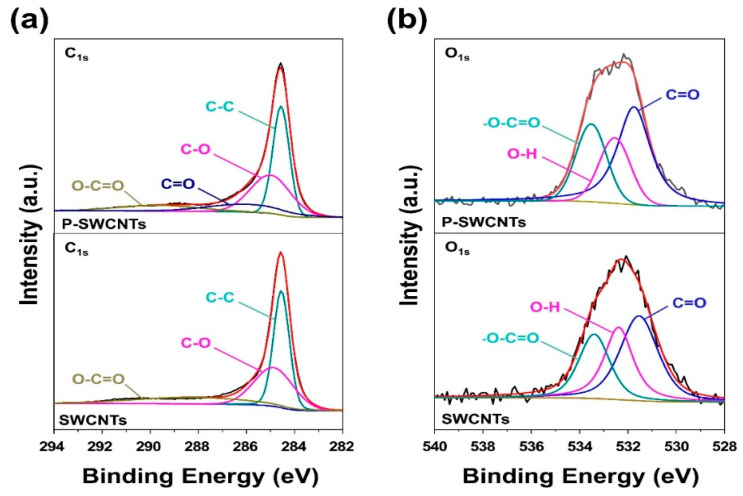
Characterization of SWCNTs and P-SWCNTs: (**a**) C_1s_ and (**b**) O_1s_ core level of XPS spectra.

In addition, the core level peaks of C_1s_ and O_1s_ for SWCNTs and P-SWCNTs are shown in Figure 2, respectively. First, as shown in Figure 2a, the electrons for the C–C, C–O, C=O, and O–C=O groups of P-SWCNTs were identified while the corresponding C_1s_ deconvoluted peak values were 284.6, 285.1, 286.4, and 288.7 eV, respectively. Secondly, the electrons for the C=O, O–H, and O–C=O groups are shown in Figure 2b while the corresponding deconvoluted peak values for O_1s_ are 531.3, 532.3, and 533.4 eV, respectively. FT-IR and XPS spectrum of SWCNTs and P-SWCNTs show efficient generation of oxygen functional groups on nano-fillers surface by atmospheric-pressure plasma treatment.

### 2.2. Morphology of Nano-Scale Fillers

**Figure 3:**
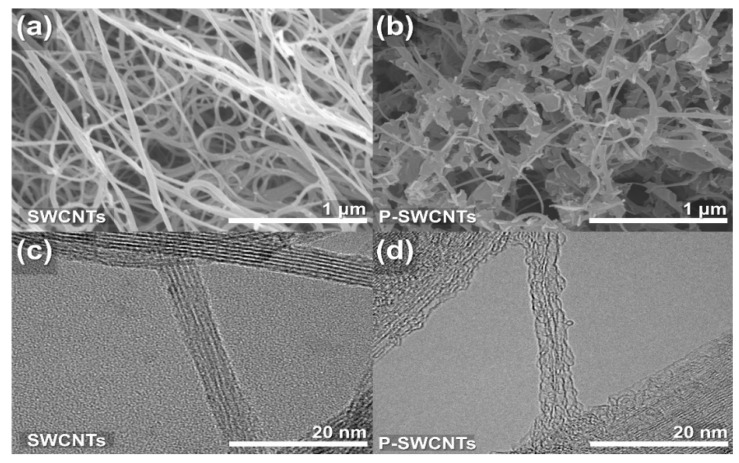
Surface morphology of SWCNTs and P-SWCNTs: (**a**,**b**) HR-SEM images and (**c**,**d**) FE-TEM.

Figure 3 shows the surface morphology of SWCNTs and P-SWCNTs. Figure 3a shows the typical arbitrary structure and high aspect ratio of SWCNTs while Figure 3b clearly shows the differences between the samples due to the damage and defects caused by surface functionalization. Furthermore, FE-TEM was used to examine the surface roughness in detail and the TEM images of the SWCNTs and P-SWCNTs surfaces are shown in Figure 3c,d, respectively. The surface of the P-SWCNTs shows clear grooves due to the etching and cleaning action of the atmospheric-pressure plasma treatment. During the atmospheric-pressure plasma treatment, successive oxidation along the sidewalls of the contaminating and amorphous carbon layers of the SWCNTs results in progressive removal of the surface.

### 2.3. Interfacial Properties of P-SCE Composites

**Figure 4:**
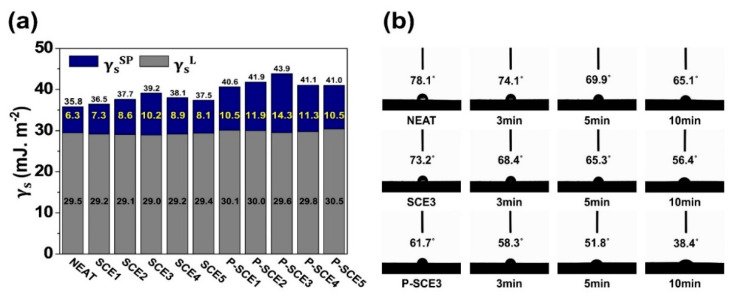
(**a**) Surface free energy and (**b**) optical image of contact angles with distilled water overtime.

The fracture behavior of the P-SCE composites is closely related to surface free energy. Here, the surface free energy is a key indicator indicating whether the interfacial adhesion with CFs is improved due to the interfacial interaction between the P-SWCNTs and the epoxy matrix. The total surface free energy can be divided into two components based on the methods of Fowkes [44], Owens [45], and Kaelble [46]. The first component is the specific polarity (Keesom interaction force, hydrogen bonding, dipole–dipole interactions, and Debye-inductive polarization) and the second is dispersion (London dispersion forces). In this experiment, the CAs between the solid and liquid were calculated to obtain the surface free energy of multi-scale composites. Moreover, the surface free energy can be obtained by the equation.

(1) $${\gamma =\gamma }^{L}+\gamma^{SP}$$

(2) $$\gamma_{L}\left(1+cos\theta \right)=2(\sqrt{\gamma_{\;S}^{L}\cdot \gamma_{\;L}^{L}}+\sqrt{\gamma_{\;S}^{SP}\cdot \gamma_{\;L}^{SP}})$$

(3) $$\frac{\gamma_{L}\left(1+cos\theta \right)}{2\sqrt{\gamma_{\;L}^{L}}}=\sqrt{\gamma_{\;S}^{SP}}\left(\frac{\sqrt{\gamma_{\;L}^{SP}}}{\sqrt{\gamma_{\;L}^{L}}}\right){+\gamma }_{\;S}^{L}$$

here γ is the total surface free energy, $\gamma^{L}$ is the dispersion component, $\gamma^{SP}$ is the specific polar component [44,47], *L* is a liquid, *S* is a solid, and *θ* is the CAs of the droplet. As mentioned above, the CAs can be obtained using three wetting liquids such as distilled water, diiodomethane, and ethylene glycol. The results of the specific polar component, dispersion component, and surface free energy of the multi-scale composites, as calculated according to Equations (1)–(3) are shown in Figure 4a, Table S2, and Figure S1. In Figure 4a, the total surface free energy of SCE composites varies from a minimum of 36.5 mJ.m^−2^ to a maximum of 39.2 mJ.m^−2^. In comparison, the total surface free energy of the P-SCE composites ranges from 40.6 mJ.m^−2^ to 43.9 mJ.m^−2^. Moreover, it was found that the minimum values for the P-SCE composites were all higher than the maximum values for the SCE composites. A similar tendency was also observed for the specific polar components. These results show that the mechanical properties can be improved by activating the interfacial interactions of the nano-scale fillers and epoxy matrix and that P-SWCNTs are more suitable to improve the interfacial interactions between CFs and epoxy matrix than untreated SWCNTs. In addition, when dividing the surface free energy of multi-scale composites into its two components, it can be seen that the specific polar components are more influential than the dispersion components. This was demonstrated by the abovementioned FT-IR and XPS results, which showed that the specific polar component influences the surface compatibility of the epoxy matrix when the hydrophobic functional groups on the surface of the P-SWCNTs are changed to hydrophilic functional groups such as carboxyl and hydroxyl groups [48,49,50].

To complement this explanation, the wetting behavior of the SCE3 and P-SCE3 composites, which exhibited the best mechanical properties, was monitored over time. The experimental conditions for experiments performed at 20 °C and for up to 10 min are shown in Figure 4b. The initial CAs of the neat composites with distilled water dropped from 78.1 to 65.1° after a maximum of 10 min. The high CAs are due to the hydrophobic groups at the interface between the CFs and the epoxy matrix. On the other hand, the initial CAs of the SCE3 composites dropped from 73.2 to 56.4° after up to 10 min, and the initial CAs of the P-SCE3 composites dropped from 61.7 to 38.4° after up to 10 min. These results show that the rich oxygen functionalities of the P-SWCNTs contribute to the enhancement of interfacial interactions with the epoxy matrix. Therefore, well-dispersed P-SWCNTs on epoxy matrix enhance the mechanical properties of multi-scale composites.

### 2.4. Mechanical Properties of P-SCE Composites

**Figure 5:**
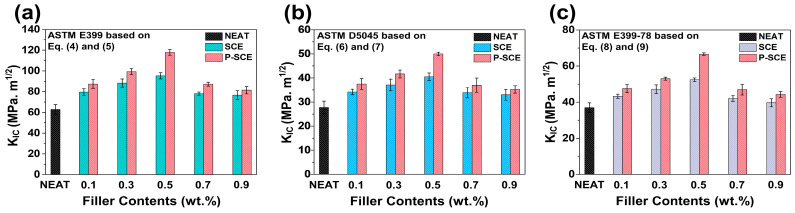
*K_IC_* comparison values of SCE and P-SCE composites according to the formula: (**a**) ASTM E399, (**b**) ASTM D5045, and (**c**) ASTM E399-78.

The potential of nano-scale fillers in multi-scale composites is a key factor in improving *K_IC_*, especially for automotive and aerospace applications where practical brittleness is essential [51,52,53,54,55]. Figure 5 shows a comparison of *K_IC_*, which represents the overall mechanical properties of SCE and P-SCE composites using each ASTM equation. First, as a result of analyzing the *K_IC_* value of the Equations (4) and (5) based on ASTM E399, the *K_IC_* value increase rates of SCE and P-SCE composites were 9.8, 12.4, 23.7, 11.7, and 6.2%, respectively. In particular, when the P-SWCNTs content was 0.5 wt.%, the *K_IC_* values were the highest in the comparison group, and when the P-SWCNTs content was 0.9 wt.%, the *K_IC_* values were the lowest. In addition, when the fillers content exceeded 0.7 wt.%, it was observed that mechanical properties rapidly decreased regardless of the presence or absence of surface modification. This means that the relatively high fillers content contributed less to the surface adhesion of multi-scale composites. In Equations (6) and (7) based on ASTM D5045 and Equations (8) and (9) based on ASTM E399-78, each increase in the *K_IC_* value shows a similar tendency to the increase in the *K_IC_* value obtained through Equations (4) and (5) based on ASTM E399. This suggests the conclusion that the tendency for the value is the same regardless of any *K_IC_* equation that can be applied to the surface-treated multi-scale composites. Besides, we compared the *K_IC_* values of multi-scale composites based on P-SWCNTs with other reported literature. Table 1 shows the improved *K_IC_* values compared to composites fabricated with various nano-fillers, suggesting that P-SWCNTs are potential reinforcements [56,57,58,59,60,61].

**Figure 6:**
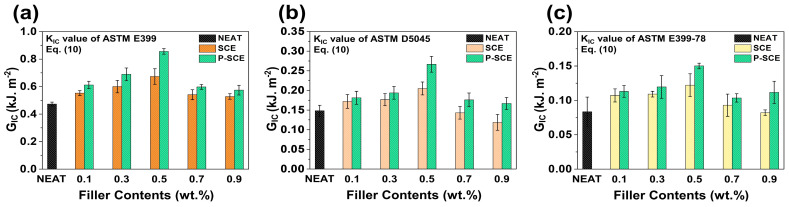
G_IC_ comparison values of SCE and P-SCE composites according to the formula: *K_IC_* value of (**a**) ASTM E399, (**b**) ASTM D5045, and (**c**) ASTM E399-78.

Figure 6 compares the G_IC_ values of SCE and P-SCE composites using *K_IC_* values of Equations (4)–(9). As expected, the values of neat composites (0.47 kJ.m^−2^) were the lowest, and the G_IC_ values of SCE composites based on *K_IC_* values of Equations (4) and (5) were SCE1 (0.55 kJ.m^−2^), SCE2 (0.60 kJ.m^−2^), SCE3 (0.67 kJ.m^−2^), SCE4 (0.54 kJ.m^−2^), and SCE5 (0.52 kJ.m^−2^), respectively. In addition, the G_IC_ values of P-SCE composites were P-SCE1 (0.61 kJ.m^−2^), P-SCE2 (0.69 kJ.m^−2^), P-SCE3 (0.86 kJ.m^−2^), P-SCE4 (0.59 kJ.m^−2^), and P-SCE5 (0.58 kJ.m^−2^), respectively. As a result, the G_IC_ increase rates of the SCE and P-SCE composites were 10.6, 14.8, 27.1, 10.3, and 8.8%, respectively. Like the *K_IC_* value, the increase rate of the G_IC_ value showed a similar tendency, and as the fillers content increased, the value decreased significantly due to the decrease in the interfacial interaction.

**Figure 7:**
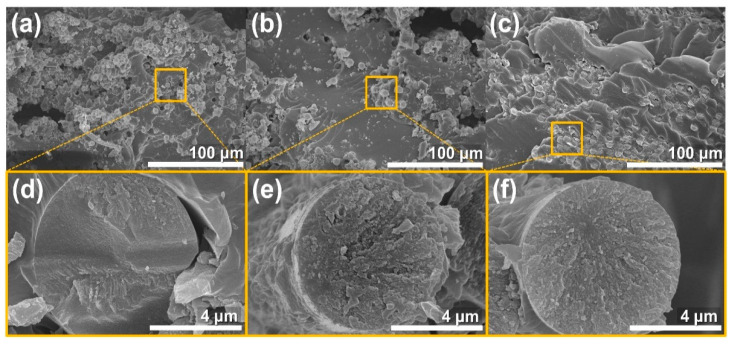
Fractured surfaces on SCE and P-SCE composites SEM images of (**a**) neat composites, (**b**) SCE3 composites, and (**c**) P-SCE3 composites: (**d**–**f**) are enlarged parts of the (**a**–**c**) image rectangles, respectively.

Figure 7 shows the SEM images of the fracture surface and fiber fracture surface for the SCE3 and P-SCE3 composites compared to the neat composites. Figure 7a is an SEM image of the neat composites showing a typical pull-out and bridging of the overall CFs and epoxy matrix. Specifically, the fracture surface of the CFs in Figure 7d exhibits a smooth fracture pattern which indicates that the fracture stresses of the CFs and epoxy matrix are not well transferred to the CFs. Figure 7b shows that the SCE3 composites exhibit a heterogeneous dispersion of SWCNTs and that less pull-out is observed compared to the neat composites. Moreover, Figure 7e shows that the fracture surface of the CFs has a rougher fracture pattern compared to the CFs of the neat composites. Due to the low interfacial adhesion between the CFs and the epoxy matrix, the fracture stress is not completely transferred to CFs. Figure 7c shows no pull-out and bridging in large-scale aggregation between the CFs and epoxy matrix of P-SCE3 composites. Besides, Figure 7f shows a very rough surface when the fracture surface of CFs is compared with Figure 7d,e. This result is because P-SWCNTs share high load even at low stress by improving interfacial adhesion and dispersibility. Therefore, intensive crack growth of CFs can be prevented, suggesting an improvement in fracture toughness.

## 3. Experimental

### 3.1. Materials

In this experiment, carbon fibers (TZ-607-12K, Taekwang, Industries Co., Seoul, Korea) based on polyacrylonitrile (PAN) was used as the reinforcing material with no sizing and surface treatment. The matrix is a diglycidyl ether of bisphenol-A (DGEBA) bifunctional epoxy resin with an epoxide equivalent weight of 185–190 g·eq^−1^, a density of 1.16 g∙m^−3^ at 25 °C, and a viscosity of 12,000 pcs (YD-128, Kukdo Chemical Co., Seoul, Korea). In addition, 4,4′-diaminodiphenylmethane curing agent (DDM, Tokyo Chemical Industry Co., Tokyo, Japan) was used. In addition, SWCNTs (Ocsial Co., Incheon, Korea) with a diameter of 1.6 ± 0.3 nm were used as fillers.

### 3.2. Atmospheric-Pressure Plasma Surface Treatment of SWCNTs

The atmospheric-pressure plasma functionalization of SWCNTs used in the manufacture of multi-scale composites is as follows: the dry oxidized surface treatment system (ATMOSMulti, PLASMART Co., Daejeon, Korea) was supplied with a high purity oxygen concentration of about 99.9%. The power for atmospheric-pressure plasma treatment of SWCNTs was 250 W, while the radio frequency was operated for 60 s at 13.5 MHz. The distance between the sample and nozzle remained constant at 3 mm.

### 3.3. Fabrication of P-SWCNTs/CFs/Epoxy Composites

**Figure 8:**
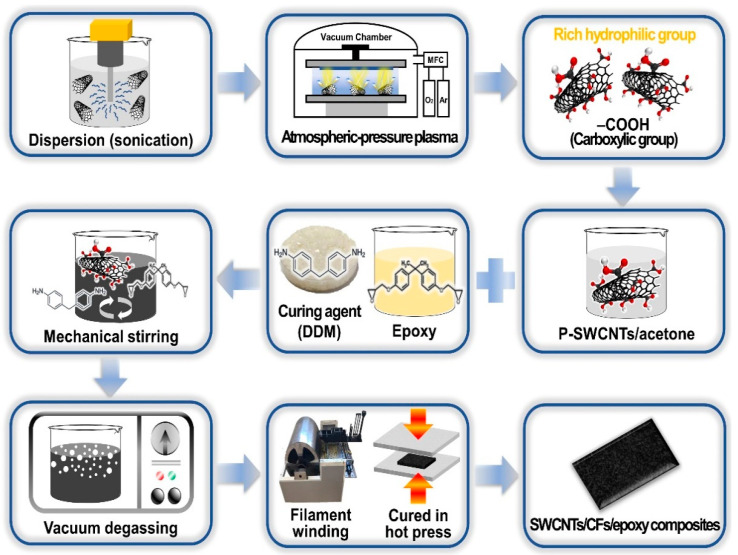
Preprocessing and preparing schematic diagram for P-SWCNTs/CFs/epoxy multi-scale composites.

P-SWCNTs/CFs/epoxy composites were prepared as shown in Figure 8: first, P-SWCNTs (0.1, 0.3, 0.5, 0.7, and 0.9 wt.%) were dispersed in acetone and sonicated for 30 min. Thereafter, the mixture was mixed with the epoxy resin and subjected to constant mechanical stirring for 40 min. Then, acetone and bubbles in the P-SWCNTs and epoxy resin mixture were removed by heating for 50 min at 100 °C in a vacuum oven. Subsequently, DDM was added to the P-SWCNTs and epoxy mixture for 5 min, followed by strong mechanical stirring. Finally, prepregs were prepared using drum windings while the laminate plates were cured at 100 °C for 30 min and at 180 °C for 2 h. We designate SWCNTs/CFs/epoxy composites as SCE and P-SWCNTs/CFs/epoxy composites as P-SCE.

### 3.4. Characterization

X-ray photoelectron spectroscopy (XPS, K-Alpha, Thermo Scientific Co., Waltham, MA, USA) and Fourier transform infrared spectroscopy (FT-IR, Vertex 80V, Bruker Co., Billerica, MA, USA) were used to analyze the surface properties of P-SWCNTs. Using a high-resolution scanning electron microscope (HR-SEM, SU8010, Hitachi Technologies Co., Tokyo, Japan) and field emission transmission electron microscope (FE-TEM, JEM-2100F, Jeol Co., Tokyo, Japan), the surfaces of multi-scale composites and P-SWCNTs were morphologically analyzed. A universal testing machine (UTM, LR-5 K Plus, Lloyd Instruments Co., West Sussex, UK) measured fracture toughness, interlaminar shear strength, and critical strain energy release rate of the multi-scale composites.

### 3.5. Testing of the P-SCE Composites

Contact angles (CAs) were measured to determine the effects of P-SWCNTs on the interfacial properties of multi-scale composites. The instrument used was a Ramé-hart CA instrument (Phoenix 300 Touch, Ramé-hart Instruments Co., Succasunna, NJ, USA) and measurements were performed using the sessile drop method. In addition, the wetting liquid used was distilled water, diiodomethane, and ethylene glycol. The measurement temperature was 20 ± 1 °C while the droplet volume was about 5 uL. CAs of multi-scale composites were measured on 15 specimens with a standard deviation of less than 1°. The surface free energy characteristics of the three wetting liquids are presented in Table S3 [62,63].

**Figure 9:**
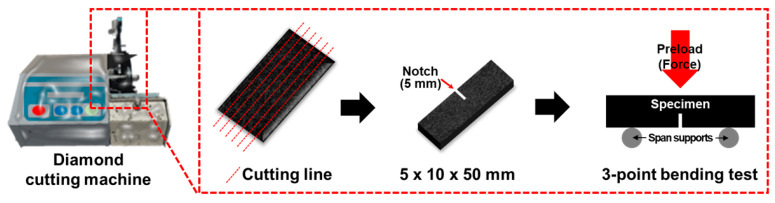
Testing details for fracture toughness (*K_IC_*) specimens.

Fracture toughness (*K_IC_*) measures the resistance of multi-scale composites to their deformation or fracture strength when subjected to short bending moments [64]. In the meantime, *K_IC_* has been used in many research methods and calculation methods in multi-scale composites. *K_IC_* comparison values are derived from metal and plastic-based equations applied to ASTM E399, ASTM D5045, and ASTM E399-78. In addition, the fracture of the specimens that derives the *K_IC_*-based value has compatibility between pre-cracking and linear elastic conditions, so all three equations were introduced. In this test, the preload is 5.6 N, the preload speed is 10 mm/min, the thickness is 5 mm, the width is 10 mm, the length is 50 mm, and the notch is 5 mm. In addition, *K_IC_* tests were performed 5 times for each specimen, and the equipment used to precision cut the composite was a diamond cutting machine (USCM, DYD-302, Daeyeong Co., Ansan-si, Korea). The approximate process of the test is detailed in Figure 9.

First, ASTM E399 is based on Equations (4) and (5).

(4) $$K_{IC}=\frac{FL}{{bd}^{3/2}}Y$$

(5) $$Y=\frac{3a/d^{3/2}[1.99-(a/d)(1-a/d)(2.15-3.93a/d)+\left(2{.7a}^{2}/d^{2}\right)]}{2(1+2a/d)(1-a/{d)}^{3/2}}$$

where *F(N)* is the critical load of specimens, *L* (mm) is the midpoint distance between the span supports, *a* (mm) is the notch length of the specimen, *b* (mm) is the width of the specimen, and *d* (mm) is the thickness of the specimen. As shown here, *Y* is the geometric element obtained from the equation.

Second, ASTM D5045 is based on Equations (6) and (7).

(6) $$K_{IC}=\frac{P}{{BW}^{1/2}}f(a/W)$$

(7) $$f\left(a/W\right)=\frac{\left(2+a/W\right)\left[0.886+4.64a/W-13.32a/W^{2}+14.72a/W^{3}-5.6a/W^{4}\right]}{{\left(1-a/W\right)}^{3/2}}$$

where *P(N)* is the critical load of specimens, *a* (mm) is the crack length of the specimen, *W* is the width of the specimen, and *B* is the thickness of the specimen. *f*(*a*/*W*) is the geometric element obtained from the equation.

Third, ASTM E399-78 is based on Equations (8) and (9).

(8) $$K_{IC}=\frac{{3PSa}^{1/2}}{2bd}Y$$

(9) $$Y=1.93-3.07\left(a/b\right)+14.53{\left(a/b\right)}^{2}-25.11{\left(a/b\right)}^{3}+25.80{\left(a/b\right)}^{4}$$

where *P(N)* is the critical load of specimens, *S* (mm) is the length of the span, and *a* (mm), *b* (mm), *d* (mm) are the notch length, width, and thickness, respectively. *Y* is the geometric element obtained from the equation.

The critical strain energy release rate (*G_IC_*) can be calculated from the *K_IC_* results and the Young’s modulus of the multi-scale composites as shown in the following Equation (10) [65].

(10) $$G_{IC}=\frac{\left({1-v}^{2}\right)K^{2}{}_{IC}}{E}$$

where *E* is Young’s Modulus from the fracture test and *v* is Poisson’s ratio (0.27) of CFs.

## 4. Conclusions

In this study, P-SWCNTs were used as nano-scale fillers to improve the interfacial adhesion of multi-scale composites. The mechanical properties of multi-scale composites were improved by enhancing the interfacial interaction between CFs and epoxy matrix. Atmospheric-pressure plasma treatments effectively enhance the oxygen content of SWCNTs and introduce the oxygen functional groups for increased interfacial interaction between fillers and matrix. Through the analysis of the surface and mechanical properties of the prepared composites, it was revealed that they have the most optimized interfacial adhesion properties when the fillers content is 0.5 wt.% regardless of surface treatments. However, the continuous increase of the fillers content showed limitations on dispersion due to the inherent van der Waals forces acting on the outer surface of SWCNTs, suggesting that there is a direct effect on the mechanical performance degradation. These results demonstrate that our method is a promising method that can improve the mechanical properties by controlling the surface of multi-scale composites based on functional groups.

## Figures and Tables

**Table 1:** Comparison of *K_IC_* improvement values by nano-fillers type in the reported literature.

Nano-Fillers	Filler Content (wt.%)	*K_IC_* Improvement (%)	References
P-SWCNTs	0.5	76	This work
SWCNT	0.1	13	[56]
Graphene	0.5	38	[57]
CNT	0.5	4	[58]
MWCNT	1.5	41	[59]
Carbon black	1.2	15	[60]
Nano-silica	4.5	34	[61]

## Data Availability

Not applicable.

## Supplementary Materials

The following are available online, Figure S1: Three wetting liquids were shown by comparing the Contact Angles value of Neat, SCE3, and P-SCE3 composites, Table S1: Surface free energy for wetting liquids, Table S2: XPS results for atomic concentrations of SWCNTs and P-SWCNTs, Table S3: Contact angles of SCE and P-SCE composites for three wetting liquids.

## Sample Availability

Samples of the compounds are not available from the authors.
