# Supplementary material for: Effect of Atmospheric-Pressure Plasma Treatments on Fracture Toughness of Carbon Fibers-Reinforced Composites
Source: Molecules. 2021 Jun 17;26(12):3698. doi: 10.3390/molecules26123698 (PMC8233790; doi:10.3390/molecules26123698)
Supplement: Supplementary file 1 [file molecules-26-03698-s001.zip › molecules-1174173-supplementary.pdf]

Supplementary Materials

Effect of atmospheric-pressure plasma treatments on fracture toughness of carbon fibers-reinforced composites

Won-Jong Kim, Young-Jung Heo, Jong-Hoon Lee, Kyong Yop Rhee[**], and Soo-Jin Park[*]

[*] *Department of Chemistry, Inha University, 100 Inharo, Incheon 22212, Republic of Korea*

[**] *Department of Mechanical Engineering, College of Engineering, Kyung Hee University, Yongin 17104, Republic of Korea*

[*] Corresponding author. Tel.: +82-32-876-7234; Fax: +82-32-867-5604.

[**] Corresponding author. Tel.: +82-31-201-2565; Fax: +82-31-202-6693.

E-mail address: sjpark@inha.ac.kr (S. -J. Park) and rheeky@khu.ac.kr (K. Y. Rhee)

Table S1. XPS results for atomic concentrations of SWCNTs and P-SWCNTs.

Samples	XPS atomic (%)		
	C_{1S}	O_{1S}	C_{1S}/O_{1S}
SWCNT	95.79	4.21	0.04
P-SWCNT	92.14	7.86	22.7

Table S2. Surface free energy for wetting liquids.

Wetting liquids	γ^{L}(mJ.m^{-2})	γ_{L}^{L} (mJ.m^{-2})	γ_{L}^{SP} (mJ.m^{-2})
Distilled water	72.80	21.80	0.38
Diiodomethane	50.80	50.42	51.00
Ethylene glycol	47.70	31.00	16.70

Table S3. Contact angles of SCE and P-SCE composites for three wetting liquids.

Specimens	Contact angles (θ)		
	Distilled water	Diiodomethane	Ethylene glycol
NEAT	78.1 ± 0.3	42.1 ± 0.2	59.8 ± 0.4
SCE1	76.2 ± 0.5	41.9 ± 0.4	58.6 ± 0.2
SCE2	73.5 ± 0.2	41.2 ± 0.3	56.3 ± 0.3
SCE3	70.2 ± 0.2	40.5 ± 0.4	53.2 ± 0.3
SCE4	72.0 ± 0.3	40.1 ± 0.1	56.7 ± 0.5
SCE5	73.8 ± 0.1	42.5 ± 0.2	54.1 ± 0.4
P-SCE1	67.9 ± 0.4	40.2 ± 0.6	44.6 ± 0.2
P-SCE2	65.6 ±0.2	39.9 ± 0.3	44.1 ± 0.2
P-SCE3	61.7 ±0.3	37.8 ± 0.1	43.1 ± 0.3
P-SCE4	66.8 ±0.5	40.1 ± 0.2	46.5 ± 0.3
P-SCE5	67.7 ±0.3	42.1 ± 0.3	42.7 ± 0.4

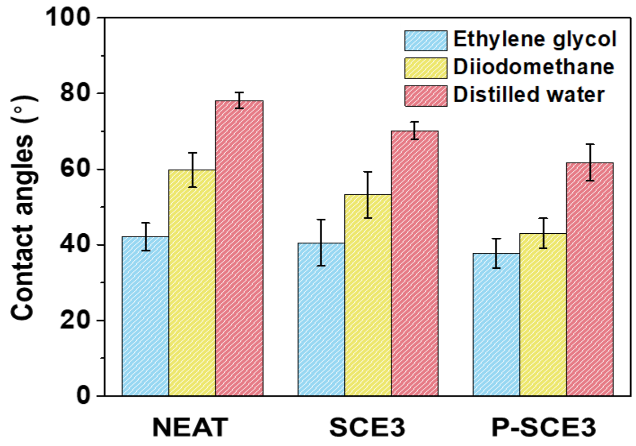

Fig. S1. Three wetting liquids were shown by comparing the contact Angles value of Neat, SCE3, and P-SCE3 composites.
